# A classification for partial subscapularis tendon tears

**DOI:** 10.1007/s00167-020-05989-4

**Published:** 2020-04-13

**Authors:** Frank Martetschläger, Frantzeska Zampeli, Mark Tauber, Peter Habermeyer, Markus Leibe

**Affiliations:** 1 Department of Shoulder and Elbow Surgery, ATOS Clinic Munich, Effnerstraße 38, 81925 Munich, Germany; 2 Helios Clinic Munich West, Department of Orthopedic Sports Medicine, Trauma Surgery and Hand Surgery, Munich, Germany; 3 Department for Orthopaedic Sports Medicine, Technical University of Munich, Munich, Germany; 4 Department of Traumatology and Sports Injuries, Paracelsus Medical University, Salzburg, Austria

**Keywords:** Subscapularis, Partial lesion, Rotator cuff, Classification

## Abstract

**Purpose:**

The aim of the study was to analyze partial subscapularis tendon (SSC) tears and provide a descriptive classification.

**Methods:**

The retrospective study included 50 patients with arthroscopically confirmed partial SSC tears. Internal rotation (IR) force measurements and IR ROM have been made and compared to the healthy contralateral side. Then the footprint of the SSC was routinely investigated by arthroscopy with standardized measurement of the bony footprint lesion. The partial tears were classified according to the mediolateral and craniocaudal extension of the rupture in the transverse and coronal plane, respectively.

**Results:**

Partial SSC tears could be classified into split lesions (type 1, *n* = 11) and 3 further groups depending on the mediolateral peeled-off length of the bony footprint (type 2: < 10 mm, *n* = 20; type 3: 10–15 mm, *n* = 10; type 4: > 15 mm, *n* = 9). Type 2–4 could be further divided depending on the craniocaudal peeled-off length of the bony footprint (group A: < 10 mm, group B: 10–15 mm, group C: > 15 mm). Significantly decreased IR strength was shown for types 2–4 (*p* < 0.05) but not for split lesions as compared to healthy side. Types 1–4 showed significant decreased active IR ROM and all except type 3 (n.s.) which showed decreased passive IR ROM compared to the healthy side (*p* < 0.05).

**Conclusion:**

We present a novel classification for partial SSC tears for a more detailed and reproducible description. This can help to improve the current knowledge about the appropriate treatment. It could be shown that partial tears of the subscapularis can have an impact on IR strength and motion.

**Level of evidence:**

III

## Introduction

Subscapularis tendon tears are more common than previously thought and have become increasingly recognized in recent years. The incidence of subscapularis tears ranges from 27 to 43% of patients undergoing shoulder arthroscopy [4, 6, 9, 26]. The subscapularis tendon is the largest rotator cuff muscle tendon unit of the shoulder and the only anterior glenohumeral stabilizer [11]. Full thickness tears affect the internal rotation of the arm leading to positive lift-off test or belly-press sign and cause pain and dysfunction of the shoulder [15, 40]. Therefore, identification of those lesions is crucial to initiate the proper therapy and improve the clinical outcome.

While complete subscapularis tears can be effectively detected during preoperative evaluation, partial subscapularis tendon tears are difficult to diagnose. MRI, ultrasound and clinical test may not provide enough information for the exact diagnosis [1, 2, 5, 11, 13, 26]. Especially smaller tears are difficult to identify with MRI preoperatively. Partial tears, graded as type 1 according to Lafosse or Fox and Romeo are diagnosed with magnetic resonance imaging only with an accuracy of 70% [14, 23, 25]. Therefore, the gold standard to identify partial subscapularis lesions remains arthroscopy [2, 5, 13].

**Fig. 1:**
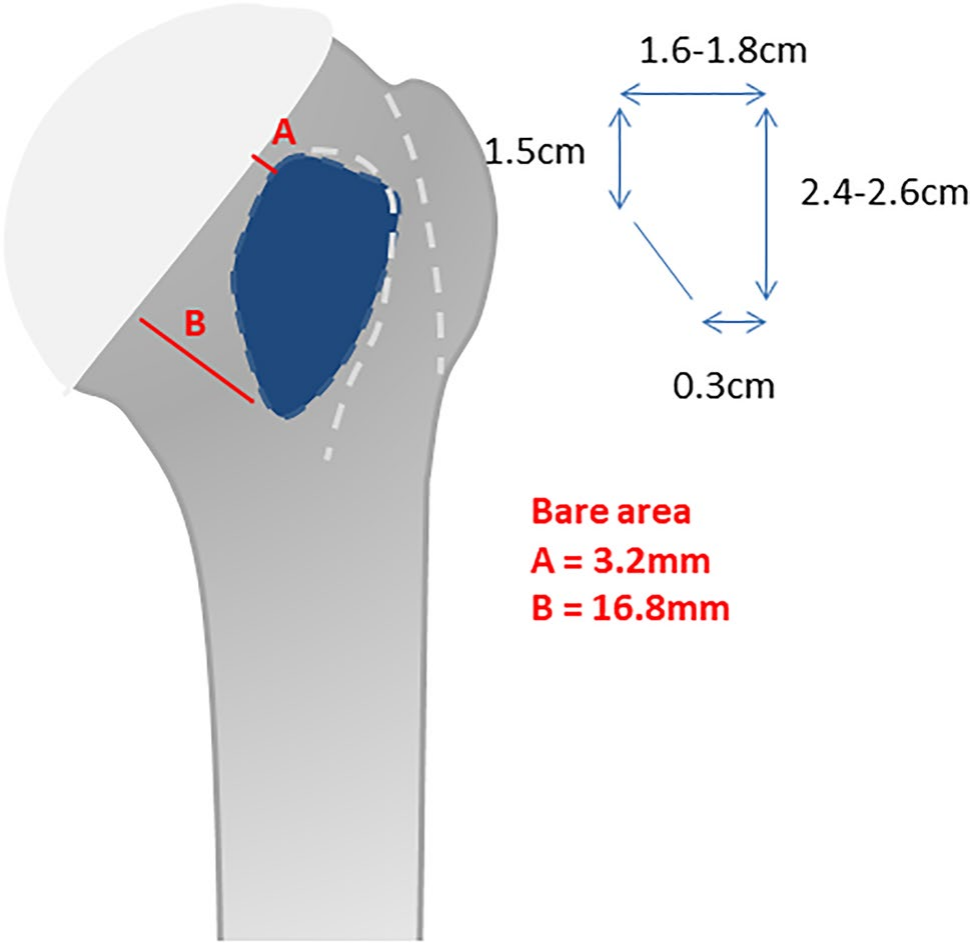
Schematic draw of the footprint of the subscapularis tendon on the lesser tuberosity. The measurements show the trapezoidal shape of the footprint at the tendon insertion site with a wider superior attachment. The mean length superior to inferior is 2.4–2.6 cm. The widest part is superior with a mean width of 1.6–1.8 cm while the most inferior aspect has a mean width of 0.3 cm. The mean width of the bare area is on the proximal part 3.2 mm and on the distal part 16.8 mm. All measurements have been proved in previous cadaveric study [19]

Understanding of the anatomy of the subscapularis footprint is crucial for an accurate diagnosis of these tears. Only knowledge of the mean size of the footprint allows a proper evaluation of the partial tear size dimension. In recent years, several studies have described the anatomy of the subscapularis tendon and its bony insertion in cadaveric specimens [19, 29, 39]. The shape of the footprint on the lesser tuberosity was described as trapezoidal at the tendon insertion site with a wider superior attachment (Fig. 1). The mean length superior to inferior was 2.45–2.63 cm. The widest part was superior with a mean width of 1.6–1.83 cm while the most inferior aspect had a mean width of 0.3 cm. Ide et al. also described the mean width of the bare area which is on the proximal part 3.2 mm and on the distal part 16.8 mm.

During shoulder arthroscopy partial subscapularis lesions can be recognized regularly. Kim et al. found 60 partial subscapularis tears during arthroscopy out of 314 patients with an incidence of 19% [22]. In a cadaveric study by Sakurai et al. approximately 50% of the shoulders showed a partial subscapularis tear at a mean age of 76 years [30]. Despite common appearance and in contrast to partial tears of the supraspinatus tendon, a distinct and particular classification for partial subscapularis tendon tears and an investigation of the clinical relevance is lacking in literature [12, 17, 34]. The tears of the subscapularis tendon have been classified according to Lafosse et al. or Fox and Romeo including all different sizes and morphologies of “partial tears” as type 1 lesions [14, 23]. By now, it is unknown whether the morphology in terms of size and tear pattern have an impact on clinical symptoms and should also be considered when choosing the treatment option. A recent study on partial SSC tears (Lafosse type 1) concluded that there is no functional or subjective benefit for repairing cranial partial tears of the SSC tendon over debridement only in the setting of an SSP reconstruction [37]. However, the term partial SSC tear may include a broad spectrum of pathology ranging from a split tear to larger partial tears and it is unknown if it is really the best option to treat all these lesions the same way.

Therefore, the aim of the present study was to specifically analyze partial lesions of the subscapularis tendon to provide a descriptive rationale to quantify partial subscapularis tears according to their morphology and to study their possible clinical relevance. This study would show if the term partial SSC tears includes different pathologies that have different impact on clinical outcome and strength. It was hypothesized that different types of partial tears of the subscapularis tendon would have an impact on the clinical symptoms of the patients and the internal rotation (IR) strength.

## Material and methods

IRB Approval has been obtained from ethical committed ATOS Klinik Heidelberg, number 01/17. Patients with arthroscopically confirmed partial tears of the subscapularis tendon from October 2016 to April 2018, were retrospectively included in the present study. Patients with previous shoulder surgery, concomitant glenohumeral osteoarthritis, or painful/injured/operated contralateral shoulders were excluded from the study. All eligible patients provided institutional review board-approved informed consent.

Fifty patients (29 male; mean age 58.5 years; range 28–76) with a partial subscapularis tear were included in the study. Sixteen patients (32%) had a history of trauma to the operated shoulder. There were 30 (60%) right shoulders and in 29 (58%) patients the dominant side was affected.

The operations were performed by a single senior surgeon specialized in shoulder surgery. During arthroscopy of the glenohumeral joint in beach chair position through standard posterior viewing portal the integrity of the footprint of the subscapularis (SSC) tendon was routinely investigated from the articular and the subcoracoid side. After debridement of potential fraying which is important to reveal the real extent of the SSC footprint lesion, measurements of the lesions on the bony footprint were performed from the anterior portal using a numeric probe with a 5 mm scale. Every lesion classified as partial subscapularis tear during arthroscopy was measured to classify the rupture more accurately. During arthroscopy, partial tear patterns were analyzed and subclassified regarding the extension of the lesion in the transverse (mediolateral extension) and coronal plane (craniocaudal extension).

**Fig. 2:**
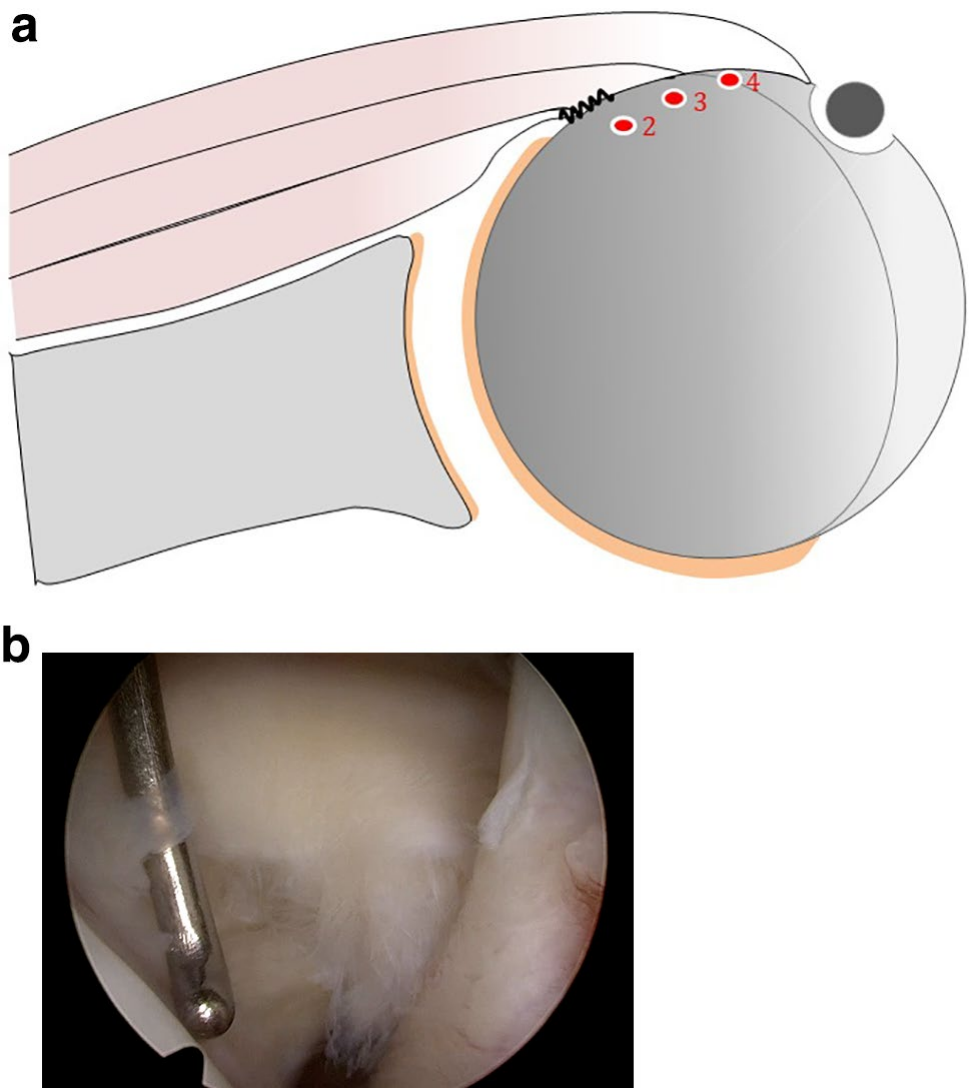
**a** Schematic draw showing the classification in the transverse plane. The main measurement is in the transverse plane and is performed at the superior border (1 cm) of the footprint, where a specific bare area of approximately 5 mm has been defined in previous studies [19]. This measurement represents the mediolateral extension of the peeled-off length of the subscapularis tendon bony footprint and started at the cartilage rim to the intact subscapularis fibers under consideration of the bare area. Type 2 represents a tear smaller than 10 mm, type 3 a measured tear 10–15 mm, and type 4 a tear larger than 15 mm. **b** Arthroscopic view (left shoulder, viewing from posterior portal) showing the measurement of the mediolateral peeled-off length of the bony footprint using a calibrated probe

The main measurement was in the transverse plane and was performed at the superior border (within 1 cm from the tendon superior edge) of the footprint, where a specific bare area with average transverse (mediolateral) distance of approximately 5 mm has been defined in previous studies (Fig. 1) [19]. This measurement represents the mediolateral extension of the peeled-off length of the SSC bony footprint and started at the cartilage rim to the intact subscapularis fibers under consideration of the bare area (Fig. 2a, b).

**Fig. 3:**
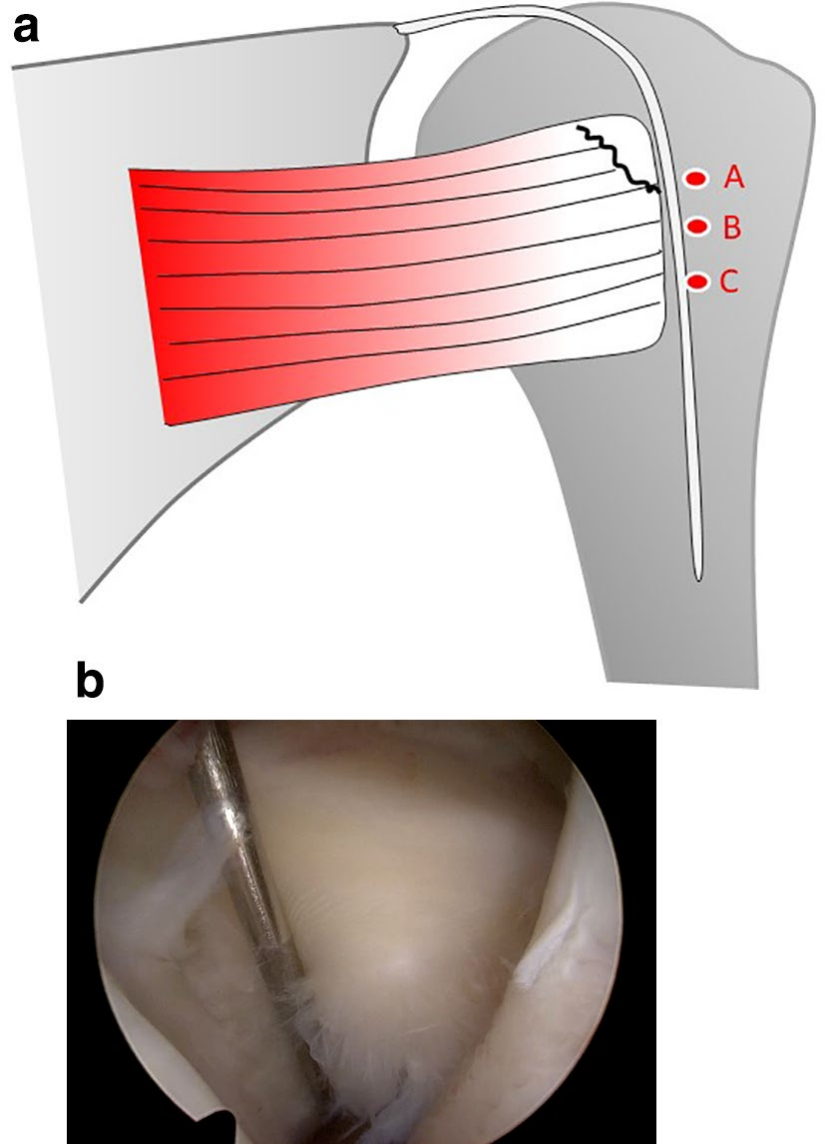
**a** Schematic draw showing the classification in the coronal plane. The secondary measurement was in the coronal plane and represents the longitudinal extension of the lesion. This was measured from cranial point of the lesion to the caudal end as the peeled-off length of the coronal bony footprint. With this measurement, types 2–4 could be further subclassified depending on the craniocaudal peeled-off length of the bony footprint. Group A is the tear length < 10 mm, group B a lesion 10–15 mm, and group C a tear larger than 15 mm. **b** Arthroscopic view (left shoulder, viewing from posterior portal) showing the measurement of the craniocaudal peeled-off length of the bony footprint using a calibrated probe

The secondary measurement was in the coronal plane and represents the craniocaudal extension of the lesion. This was measured from the most cranial point of the lesion to the caudal end as the peeled-off length of the bony footprint (Fig. 3a, b). Both main and secondary measurements was done with 1 mm accuracy. Two specialized shoulder surgeons (FM, MT), evaluated the intraoperative recordings (videos). During the estimation and to test the inter- and intra- observer agreement of the classification, it was necessary to stop the video at the point that the observer thinks it is the best image for measuring the detected lesions as if he would do in real surgery conditions and of course to avoid looking the following part where the measurement from the surgeon was carried out. The classification of the tear was made by the observer by his experience and by using the screen measurement tool. The intraobserver and interobserver agreement was assessed by intraclass correlation coefficients (ICCs). For intraobserver reliability, one of the surgeons (FM) evaluated again all intraoperative recordings after 1 month.

Concomitant lesions of the biceps pulley and/or the supraspinatus tendon were also recorded [17, 18].

Preoperatively, all patients underwent clinical evaluation for subscapularis (belly-press test, lift-off test), supraspinatus and long head of the biceps tendon pathologies. Active and passive IR was evaluated for both injured and contralateral healthy side and was graded as: (1) buttock; (2) sacroiliac joint; (3) 5th lumbar vertebra (L5); (4) 1st lumbar vertebra (L1); (5) 7th thoracic vertebra (T7). Pain was evaluated during belly-press and lift-off test using the VAS scale. In addition, force measurements for IR were performed in the bear-hug position and compared to the pain free, healthy (checked with ultrasound) contralateral side. Maximal isometric voluntary contraction (MVC) was measured with a MicroFet 2 hand-held dynamometer which is an accurate, portable Force Evaluation and Testing (FET) device, with a sample frequency of 10 sample/s (Hogan Health Industries, Inc. 8020 South 1300 West, West Jordan, USA) [32, 33].

A pilot study of twelve healthy individuals was carried out for IR strength with the same instrumentation and at the same testing position to detect any differences between dominant and non-dominant side for this setup. Twelve healthy volunteers (6 male; mean age 38.8 years; SD 9.9; range 25–51, dominant side 9 right, 1 left) with no previous shoulder injury or surgery, no glenohumeral osteoarthritis, and pain-free shoulder joints for both sides were studied. The measurements were performed by one experienced tester with over 5 years of experience performing muscle strength measurement with hand-held dynamometry (HHD). The measurements were performed three times and the mean value was used for analysis. The order of testing side (dominant, non-dominant), was randomly selected.

### Statistical analysis

Descriptive statistics were used to evaluate the distribution of types of SSC partial tears. To detect differences of IR force and IR ROM between the injured shoulder and the contralateral healthy side we used the Wilcoxon signed rank test. Intraobserver and interobserver reliability regarding the tear pattern of subscapularis tear was determined by ICCs with 95% confidence intervals (Cis). The agreement was classified as poor (< 0.4), moderate (0.4–0.8), or excellent (> 0.8). All statistical analyses were performed with SPSS, version 19.0 for Windows (IBM Corp, Ehningen, Germany). The level of significance was set at *p* < 0.05. Post hoc power analysis was performed when results did not reach statistical significance level.

## Results

### Concomitant pathologies

Descriptive statistics for incidence of concomitant pathologies are described in Table 1.

**Table 1:** Concomitant lesions

	Full cohort	Partial subscapularis tear			
		Type I	Type II	Type III	Type IV
Supraspinatus tear (SS)	40 (80%)	7 (63%)	17 (85%)	9 (90%)	7 (78%)
Full thickness SS tear	20	2	8	6	4
Partial thickness SS tear	20	5	9	3	3
LHBT tendinitis	49 (98%)				
SLAP lesion	18 (36%)				
Pulley lesion^a^	50 (100%)				
Type I	0				
Type II	2 (4%)				
Type III	8 (16%)				
Type IV	40 (80%)				

Results given in absolute numbers and in parenthesis the percent of full cohort of patients

*LHBT* long head biceps tendon, *SLAP* superior labrum from anterior to posterior

^a^According to Habermeyer et al. [18]

### Tear pattern

**Fig. 4:**
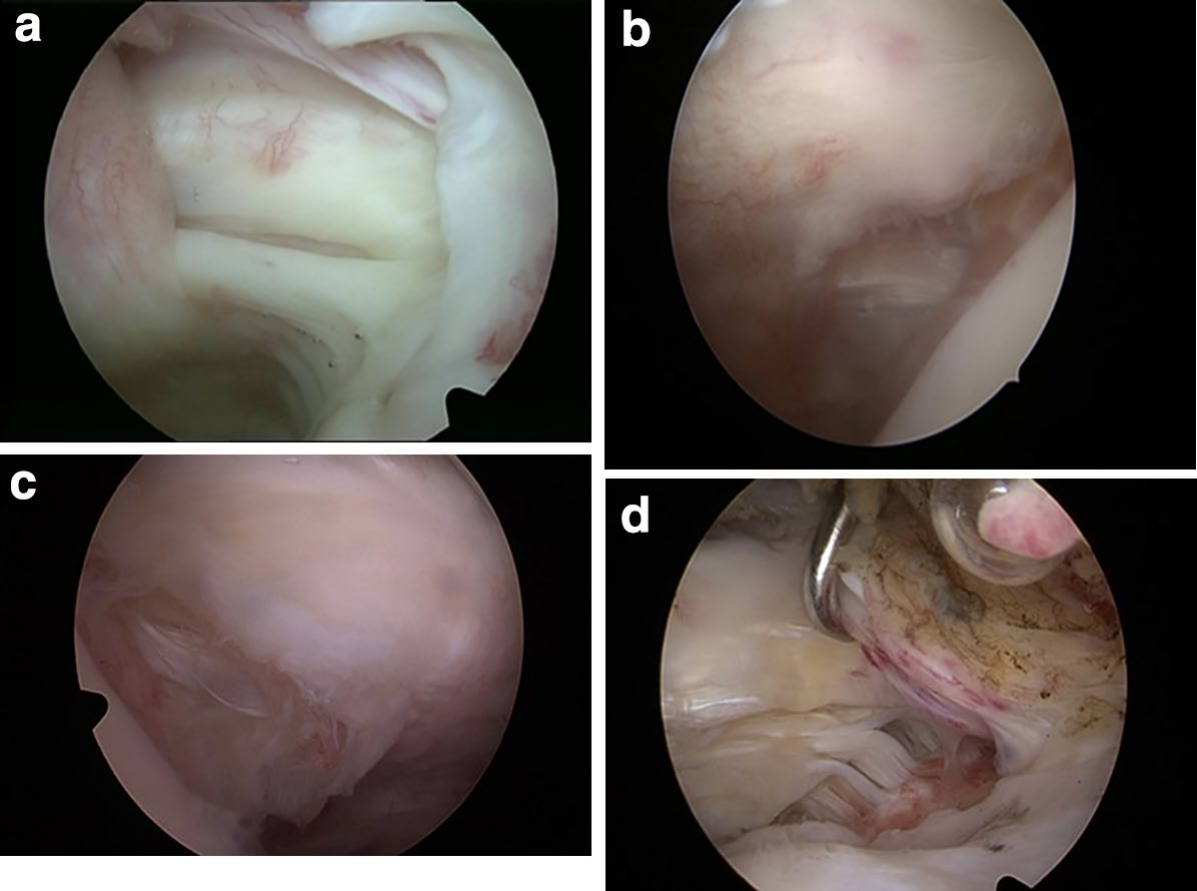
Tear pattern for partial subscapularis tear according to the mediolateral peeled-off length of the bony footprint taken in consideration the bare area (5 mm). **a** Split lesion is classified as type 1. **b** Type 2 tear represents a tear smaller than 10 mm. **c** Type 3 represents a tear 10–15 mm. **d** Type 4 a tear larger than 15 mm

According to the measured dimensions of the peeled-off length of the SSC bony footprint we described the tear patterns. The partial subscapularis tendon tears could be classified into split lesions (type 1; Fig. 4a) and three different groups depending on the mediolateral peeled-off length of the bony footprint (Fig. 2a). Type 2 represents a tear smaller than 10 mm (Fig. 4b), type 3 a measured tear 10–15 mm (Fig. 4c), and type 4 a tear larger than 15 mm (Fig. 4d). Types 2–4 could be further subclassified depending on the craniocaudal peeled-off length of the bony footprint. Group A is the tear length < 10 mm, group B a lesion 10–15 mm, and group C a tear larger than 15 mm (Fig. 3a).

### Tear pattern distribution

There were 11 type 1 (22%), 20 type 2 (40%), 10 type 3 (20%) and 9 type 4 lesions (18%). The results for coronal sub-classification and descriptive statistics for subscapularis tendon tear pattern distribution are depicted in Table 2. The intraobserver and interobserver agreement for the tear pattern including both types (1–4) and subtypes (A–C), as assessed by ICCs, was excellent [0.92 (95% CI 0.9–0.94) and 0.9 (95% CI 0.89–0.91), respectively].

**Table 2:** Frequencies (relative frequencies in %) of types and subtypes of partial subscapularis tears according to the proposed classification

Transverse plane (mediolateral extension)	Coronal plane (craniocaudal extension of tear)			
	Subtype A < 10 mm	Subtype B 10–15 mm	Subtype C > 15 mm	
Type 1: split	11			11 (22%)
Type 2: < 10 mm	7 (14%)	6 (12%)	7 (14%)	20 (40%)
Type 3: 10–15 mm		4 (8%)	6 (12%)	10 (20%)
Type 4: > 15 mm		2 (4%)	7 (14%)	9 (18%)

### Clinical tests and force measurement

Twenty-six (52%) patients showed a positive lift-off test and 21 (42%) a positive belly-press test. The mean VAS during the lift-off test was 3.3 and 2.7 during the belly-press test. Table 3 shows the results of the clinical tests and the VAS for the types 1–4 and for their subtypes (A–C) (Table 3).

**Table 3:** Frequencies of patients with positive lift-off and belly-press test according to the type-subtype tear pattern

	Subtype	Lift-off test Positive	Belly-press test Positive	Mean VAS lift off	Mean VAS belly press
Type 1		6	2	3.3	1.4
		54.5%	18.2%		
Type 2	A	4	4	3.1	1.7
	B	2	3	2.3	3.0
	C	3	1	1.9	2.7
		45%	40%	2.4	2.4
Type 3	A				
	B	2	3	2.3	5.0
	C	3	3	4.5	4.0
		50%	60%	3.8	4.3
Type 4	A				
	B	2	1	4.0	0.5
	C	4	4	4.0	3.6
		66.7%	55.6%	4.0	2.9

Relative frequency of positive tests for each type of tears (1–4). Mean values of pain measured in visual analogue scale (VAS) during lift-off and belly-press test. Mean value of VAS score for each type (1–4) is also given

Patients with partial subscapularis tears showed a significantly decreased IR strength (mean 15.4 Nm; range 4.7–28; SD 6.1) when compared to the healthy contralateral side (18.7 Nm; range 5.9–34; SD 6.8; *p* < 0.000). The decrease of IR strength was detected irrespectively of coexistence or not of supraspinatus (SSP) tear (either partial or complete). More specifically, there was significant IR strength decrease as compared to contralateral healthy side for the isolated partial SSC group (injured; mean 13 Nm; range 4.7–27; SD 6.4, vs healthy; mean 17.4 Nm; range 8.2–24; SD 5.1; *p* = 0.022), for the group with partial SSC and concomitant partial SSP tear (injured; mean 16.3 Nm; range 8.2–26.6; SD 5.9, vs healthy; mean 20.5 Nm; range 9.6–30; SD 6.2; *p* = 0.001) and for the group with partial SSC and complete SSP tear (injured; mean 15.7 Nm; range 7.4–28; SD 6.1, vs healthy; mean 17.7 Nm; range 5.9–34; SD 7.9; *p* = 0.017). When evaluating each group of partial SSC tears separately, patients with tears type 2, 3 and 4 showed significantly decreased IR strength (*p* 0.004, 0.022, and 0.008, respectively), while patients with type 1 (split lesions) did not show significant difference from healthy side (n.s.). For the non-significant result, a post hoc power analysis for Wilcoxon signed ranked tests (matched pairs), with alpha 0.05, sample size 11, and large effect size for difference in force (*d* = 0.8) showed 1 − *β* (power) 0.93. The results of force measurements for each type are shown in Table 4. A pilot study of 12 healthy individuals showed no significant side-to-side difference for internal rotation strength measurement using the same setup (mean 33.3 Nm; range 17.2–60; SD 13.7 vs mean 33.6 Nm; range 16.6–52.7; SD 12.5, n.s.). For this comparison the effect size was small *d* = 0.13 showing no clinically significant difference between dominant and non-dominant side. The post hoc power analysis for this comparison with small effect size *d* = 0.13, *a* = 0.05, sample size 12, showed 1 − *β* = 0.15.

**Table 4:** Force measurements for internal rotation expressed in Nm

Mean force (Nm) (range; SD)	Injured side	Healthy side	*p* value
Type 1	13.9 (5.4–22; 4.5)	17.0 (10–21; 3.2)	n.s
Type 2	15.6 (7.4–28; 6.4)	18.2 (6.4–30; 7.2)	0.004
Type 3	14.5 (4.7–25.7; 6.7)	17.3 (5.9–29; 8.1)	0.022
Type 4	17.7 (8.7–26.6; 6.3)	23.6 (12.8–34; 6.3)	0.008

*n.s.* non-significant (> 0.05), *SD* standard deviation

There was significantly decreased IR ROM both active and passive when compared to the healthy contralateral side (*p* 0.000). When evaluating each group separately (types 1–4), we found significant decreased IR compared to contralateral side both active and passive for all comparisons except from passive ROM for group type 3 (n.s.) (Table 5). For the non-significant result, a post hoc power analysis for Wilcoxon signed ranked tests (matched pairs), with alpha 0.05, sample size 10, and large effect size for difference in IR (*d* = 0.9) showed 1 − *β* 0.89.

**Table 5:** Internal rotation ROM measurements

Mean IR (SD; range)	Injured side	Healthy side	*p* value
Type 1			
Active	3.6 (0.9; 2–5)	4.5 (0.5; 1–5)	0.007
Passive	3.9 (0.9; 2–5)	4.5 (0.5; 1–5)	0.034
Type 2			
Active	3.7 (1.0; 2–5)	4.4 (0.8; 3–5)	0.018
Passive	3.8 (0.9; 2–5)	4.4 (0.8; 3–5)	0.013
Type 3			
Active	4 (1.0; 2–5)	5 (0; 5–5)	0.026
Passive	4.2 (1.1; 2–5)	5 (0; 5–5)	n.s
Type 4			
Active	3.2 (1.4; 1–5)	4.8 (0.4; 4–5)	0.016
Passive	3.6 (1.2; 1–5)	4.8 (0.4; 4–5)	0.016

The IR was graded as: 1, buttock; 2, sacroiliac joint; 3, 5th lumbar vertebra (L5); 4, 1st lumbar vertebra (L1); 5, 7th thoracic vertebra (T7)

*n.s.* non-significant (> 0.05)

## Discussion

The most important finding of the study was that partial subscapularis tears can be clearly classified in four different types. While tendon insertion can be intact in type 1 lesions, showing a horizontal split tear, the remaining cases showed a different amount of articular-sided tendon detachment (types 2–4) in the important proximal zone, nowadays called the “leading edge” of the tendon [11]. This was reflected in the results as in type 1 (split lesions) no differences from healthy side were detected for strength testing, while types 2–4 showed significant differences from contralateral healthy side regarding the internal rotation strength and the active and passive IR motion and therefore, the study hypothesis was confirmed. This new classification system for partial tears of the subscapularis is described to provide better comparability and investigation of these lesions in the future.

Subscapularis tendon tears are common lesions of the shoulder and can lead to ongoing shoulder pain and impairment of shoulder function. While full-thickness tears have been widely investigated over the years, morphology and clinical relevance of partial subscapularis tears have not been intensively investigated yet [1, 4, 7, 8, 15]. While there are several studies describing different classification systems and the optimal treatment strategies for partial supraspinatus tendon tears, literature is lacking a vast number of similar studies regarding the subscapularis tendon [3, 12, 17, 21, 24, 28, 31, 35, 36, 38]. This might be due to the available classification systems for subscapularis tears that do not allow for a clear sub-classification of partial tears. The classification systems published by Fox and Romeo and Lafosse et al. summarize all partial tears as type 1 lesions, not allowing for a clear differentiation [14, 23]. In a recent study that investigated partial SSC tears Lafosse type 1, it was concluded that there was no functional or subjective benefit of repairing cranial partial tears of the SSC tendon over debridement only in the setting of an SSP reconstruction in a 24 months follow-up [37]. However, it is unknown if this lack of benefit may be due to the inclusion of several types of partial tears, or if the two groups of that study have similar incidence of different types of partial SSC tears. In their classification for subscapularis tears, Yoo et al. proposed a sub-classification for partial tears regarding the coverage of the footprint with four facets A–D; type 1 lesions fraying or longitudinal split lesions, type 2 tears were classified a tear with less (2A) or greater than 50% (2B) of the first facet of the tendon detachment from the footprint [40]. However, during arthroscopy it might be difficult to identify the four different facets. In the present study, the split lesions with intact footprint coverage showed to have different impact on clinical shoulder function impairment compared to all other partial tears, which showed a partial detachment of the tendon from the footprint. Therefore, we propose to differentiate between these morphologies.

Regarding the clinical relevance of partial subscapularis lesions, it was shown that even partial tears might affect IR strength and lead to painful impairment and positive clinical tests. While type 1 (split lesions) did not show significant differences in IR strength, types 2–4 showed significant decrease for IR strength compared to the healthy side. This could not be shown in a pilot study on ten healthy individuals. Furthermore, both active and passive IR motion was decreased for groups 1–4 as compared to healthy side. To our knowledge, the clinical relevance of partial subscapularis lesions has not been investigated widely yet. For the subclassifications A–C we did not carry any further comparisons due to the low number of cases for the subgroups A–C for each main group 2–4. Since the extension of the tears in the coronal plane tend to correlated with tear size in the transverse plane, we believe the sub-classification in the coronal plane (A, B, C) to be less important for classification in daily clinical practice. Therefore, for practical reasons, we suggest using types 1–4 only.

In a recent study, Katthagen et al. examined a single suture anchor repair of upper third complete and partial isolated tears of the subscapularis tendon [20]. In their series, partial lesions have been repaired when involving more than 50% of the coronal footprint coverage. For both, complete and partial tears they found excellent clinical outcomes with improves function and pain reduction. These results are in accordance with the results of the present study, underlining that even partial tears can lead to clinical problems in terms of pain and function and warrant surgical treatment.

As known from complete subscapularis tears, isolated lesions are rare [10, 27, 38]. This also applies to partial lesions of the subscapularis. In the presented cohort there were only 20% of isolated lesions while 80% showed concomitant lesions of the supraspinatus tendon. All patients (100%) had concomitant lesions of the biceps pulley sling according to Habermeyer classification [18]. These results highlight the importance of a clear investigation of the long head of the biceps tendon (LHB) in patients with partial subscapularis tears. In patients with LHB instability an isolated repair of the subscapularis might result in ongoing biceps instability and pain. Therefore, the authors recommend performing a tenotomy or tenodesis of the LHB for patients with additional biceps instability. These findings do correlate with the presented results by Godeneche et al. who also stated these lesions to merit special recognition since they might destabilize the LHB [16].

Finally, some limitations of the study need to be mentioned. It is possible that a force difference exists between the dominant and the non-dominant shoulder for the internal rotation, however, the effect size of the comparison between two sides of a pilot study was small (*d* = 0.13). Also, it should be noted that 80% of the patients had concomitant lesions of supraspinatus and 100% lesions of pulley sling that might have influenced especially the pain values. However, it was shown IR strength was significantly decreased irrespectively of coexistence or not of a supraspinatus tear. Finally, although there have been reported differences between larger/male and smaller/female specimens regarding the SSC tendon bony footprint, however the dimensions of the bare area at the proximal end have shown consistent measurements irrespectively of specimen size [19].

However, this is the first study in literature giving an easy and reliable measurement tool and classification for all partial lesions of the subscapularis tendon according to their detachment from the footprint along with clinical data, investigating their potential relevance of these lesions. The precise collection of all concomitant lesions can help to better understand and identify these pathologies of the anterior shoulder compartment. The clinical relevance of this study is that the term partial SSC tear includes a broad spectrum of pathology ranging from a split tear to larger partial tears and not all partial SSC tears are the same. This was also reinforced by the different impact they had on internal rotation strength measurements.

## Conclusion

A novel classification for partial SSC tears is presented for a simple but more detailed and reproducible description. It could be shown that partial tears of the subscapularis can have different impact on IR strength and motion and that not all partial SSC tears are the same.
